# Quantifying the effects of antiangiogenic and chemotherapy drug combinations on drug delivery and treatment efficacy

**DOI:** 10.1371/journal.pcbi.1005724

**Published:** 2017-09-18

**Authors:** Sirin Yonucu, Defne Yιlmaz, Colin Phipps, Mehmet Burcin Unlu, Mohammad Kohandel

**Affiliations:** 1 Department of Physics, Bogazici University, Bebek, Istanbul, Turkey; 2 Center for Life Sciences and Technologies, Bogazici University, Bebek, Istanbul, Turkey; 3 School of Pharmacy, University of Waterloo, Waterloo, Ontario, Canada; 4 Department of Applied Mathematics, University of Waterloo, Waterloo, Ontario, Canada; University of California Irvine, UNITED STATES

## Abstract

Tumor-induced angiogenesis leads to the development of leaky tumor vessels devoid of structural and morphological integrity. Due to angiogenesis, elevated interstitial fluid pressure (IFP) and low blood perfusion emerge as common properties of the tumor microenvironment that act as barriers for drug delivery. In order to overcome these barriers, normalization of vasculature is considered to be a viable option. However, insight is needed into the phenomenon of normalization and in which conditions it can realize its promise. In order to explore the effect of microenvironmental conditions and drug scheduling on normalization benefit, we build a mathematical model that incorporates tumor growth, angiogenesis and IFP. We administer various theoretical combinations of antiangiogenic agents and cytotoxic nanoparticles through heterogeneous vasculature that displays a similar morphology to tumor vasculature. We observe differences in drug extravasation that depend on the scheduling of combined therapy; for concurrent therapy, total drug extravasation is increased but in adjuvant therapy, drugs can penetrate into deeper regions of tumor.

## Author summary

Tumor vessels being very different from their normal counterparts are leaky and lack organization that sustains blood circulation. As a result, insufficient blood supply and high fluid pressure begin to appear inside the tumor which leads to a reduced delivery of drugs within the tumor, especially in tumor center. A treatment strategy that utilizes anti-vascular drugs is observed to revert these alterations in tumor vessels, making them more normal. This approach is suggested to improve drug delivery by enhancing physical transport of drugs. In this paper, we build a mathematical model to simulate tumor and vessel growth as well as fluid pressure inside the tumor. This framework enables us to simulate drug treatment scenarios on tumors. We use this model to find whether the delivery of the chemotherapy drugs is enhanced by application of anti-vascular drugs by making vessels more normal. Our simulations show that anti-vascular drug not only enhances the amount of drugs that is released into tumor tissue, but also enhances drug distribution enabling drug release in the central regions of tumor.

## Introduction

The abnormal structure of tumor vasculature is one of the leading causes of insufficient and spatially heterogeneous drug delivery in solid tumors. Tortuous and highly permeable tumor vessels along with the lack of a functional lymphatic system cause interstitial fluid pressure (IFP) to increase within tumors. This elevated IFP results in the inefficient penetration of large drug particles into the tumor, whose primary transport mechanism is convection [1, 2].

The abnormalities in tumor vasculature are caused by dysregulation of angiogenesis. Tumors initiate angiogenesis to form a vascular network that can provide oxygen and nutrients to sustain its rapid growth. The production of VEGF, a growth factor that promotes angiogenesis, is triggered by the chronic hypoxic conditions that are prevalent in tumors. Besides inducing angiogenesis, it leads to hyperpermeable blood vessels by enlarging pores and loosening the junctions between the endothelial cells that line the capillary wall [3, 4]. Subsequently, excessive fluid extravasation from these vessels results in a uniformly elevated IFP in the central region of tumor nearly reaching the levels of microvascular pressure (MVP) while at the tumor periphery, IFP falls to normal tissue levels [1, 5, 6]. This common profile of IFP within tumors has been identified as a significant transport barrier to therapeutic agents and large molecules [1, 7]. When IFP approaches MVP, pressure gradients along vessels are diminished and blood flow stasis occurs, diminishing the functionality of existing vessels [8–10]. Furthermore, uniformity of IFP in interior regions of tumors terminates the convection within tumor interstitium, hindering the transportation of large drugs [1]. While the lack of a transvascular pressure gradient inhibits convective extravasation of drugs, sharp IFP gradient at tumor periphery creates an outward fluid flow from tumors that sweeps drugs away into normal tissues [1]. Together these factors lead to the decreased drug exposure of tumor cells.

It has been revealed that the application of antiangiogenic agents can decrease vessel wall permeability and vessel density, transiently restoring some of the normal function and structure of abnormal tumor vessels [4, 11, 12]. This process, which is called vascular normalization, is associated with a decrease in IFP and an increase in perfusion. Therefore, this state of vasculature enables increased delivery of both drug and oxygen/nutrients to the targeted tumor cells [11, 13]. Normalization enhances convection of drug particles from vessels into tumor interstitium by restoring transvascular pressure gradients through IFP reduction [11, 14, 15]. It has shown some favorable results in preclinical and clinical trials regarding the enhancement of the delivery of large therapeutics such as nanoparticles [14, 16, 17]. Since nanoparticles benefit from the enhanced permeability and retention effect (EPR), they are distributed in higher amounts to tumors relative to normal tissue. Accumulation of nanoparticles in normal tissues is relatively small compared to the standard small molecule chemotherapies, leading to decreased toxicity and side effects. However, the main transport mechanism for large drugs is convection in tumor microenvironment. Hence, when IFP is high, extravasation via convection is inhibited. Normalization due to its ability to decrease IFP seems promising in drug delivery for large drugs with its potential of restoring convective transportation.

In both clinical and preclinical studies, it has been shown that antiangiogenic drugs demonstrate anti-tumor effects in various cancer types [18]. However, rather than using antiangiogenic agents alone, studies reveal that the combination of these agents with chemotherapy drugs yields favorable results with increased therapeutic activity. In some clinical studies [19–21], bevacizumab combined with conventional chemotherapy has increased the survival and response rates among patients with gastrointestinal cancer compared to bevacizumab alone. This finding that antiangiogenic therapy in combination with chemotherapy can improve the efficacy of treatment has been observed for patients with various cancers including non-small cell lung cancer [22, 23], breast cancer [24–26] and ovarian cancer [27]. However, it is evident that there is a transient time window for vessel normalization [28, 29]. In order to improve drug delivery, chemotherapy should coincide with this transient state of improved vessel integrity. Prolonged or excessive application of antiangiogenic agents can reduce microvascular density to the point that drug delivery is compromised [30]. Therefore, dosing and scheduling of combined therapy with antiangiogenic agents must be carefully tailored to augment the delivery and response to chemotherapy [12]. It is suggested that rather than uninterrupted application, intermittent cycles which can create re-normalization should be employed for antiangiogenic agent scheduling [31].

Due to the complex and interdisciplinary nature of the subject, there is a considerable amount of computational efforts on tumor vascularization and its consequences for the tumor microenvironment and drug delivery. Development of vasculature and intravascular flow dynamics are studied comprehensively [32–37] and in many studies chemotherapy is given through the discrete vessel system in order to calculate drug delivery to capillaries and tumor [33, 34, 37–39]. Mathematical models have included transvascular and interstitial delivery of drugs [37–39]. In addition to that, Wu et al. added tumor response to chemotherapy by applying nanoparticles and evaluating the decrease in tumor radius during chemotherapy for different microenvironmental conditions [39]. There are also some studies about the optimization of combination therapy in tumors [40]. In studies by the groups of Urszula Ledzewicz and Heinz Schäettler, changes in tumor volume after the administration of cytotoxic and antiangiogenic agents have been investigated by proposing a mathematical model and seeking optimal solutions for different treatment cases [41, 42]. Compartment models have also been used to explore how antiangiogenic agents may provide assistance to chemotherapy agents in reducing the volume of drug-resistant tumors and by using a bifurcation diagram it is shown that the co-administration of antiangiogenic and chemotherapy drugs can reduce tumor size more effectively compared to chemotherapy alone [43].

Applications of chemotherapy drugs together with antiangiogenic agents have been studied by Panovska et al. to cut the supply of nutrients [44]. Stephanou et al. showed that random pruning of vessels by anti-angogenic agents improves drug delivery by using 2-D and 3-D vessel networks [45]. However, they did not associate this benefit with normalization of vasculature. Jain and colleagues laid out the general groundwork for relations between vessel normalization and IFP by relating vessel properties and interstitial hydraulic conductivity to changes in pressure profile due to normalization [15]. The subject is further investigated by Wu et al. by building a 3-D model of angiogenesis and adding intravascular flow to the computational framework [32]. They observed slow blood flow within the tumors due to almost constant MVP and elevated IFP profile. They show the coupling between intravascular and transvascular flux. Kohandel et al. showed that normalization enhances tumor response to chemotherapy and identified the most beneficial scheduling for combined therapy in terms of tumor response [46]. The size range of nanoparticles that could benefit from normalization has also been investigated [16].

In this study, following the continuous mathematical model developed by Kohandel *et al*. [46] which couples tumor growth and vasculature, we built a framework for tumor dynamics and its microenvironment including IFP. We use this system to evaluate the improvement in nanoparticle delivery resulting from vessel normalization.

As the tumor grows, a homogeneous distribution of vessels is altered by the addition of new leaky vessels to the system, representing angiogenesis. As a consequence of angiogenesis and the absence of lymph vessels, IFP starts to build up inside the tumor inhibiting the fluid exchange between vessels and tumor and inhibiting nanoparticle delivery. Simulations give the distribution of the nanoparticles in the tumor in a time-dependent manner as they exit the vessels and are transported through interstitium. The activity of the drugs on tumor cells is determined according to the results of experimental trials by Sengupta et al. [47]. We apply drugs in small doses given in subsequent bolus injections. During drug therapy, both vessels and tumor respond dynamically. After injections of antiangiogenic agents, a decrease in vessel density accompanies the changes in vessel transport parameters, initiating the normalized state.

Combining chemotherapy with applications of antiangiogenic agents, we are able to identify the benefits of a normalized state by observing the effects of different scheduling on IFP decrease, extravasation of drugs and tumor shrinkage. We found that in adjuvant combination of drugs, IFP and vessel density decrease together resulting in an increase in the average extravasation of nanoparticles per unit area in the interior region of tumor. In concurrent combination of drugs, IFP decrease is higher but vessel decrease is higher as well, creating a smaller enhancement in average extravasation per unit tumor area. However, even though average extravasation is smaller in this case, we observe an increase in homogeneity in drug distribution. Nanoparticles begin to extravasate even in the center of tumor through sparsely distributed vessels due to the sharp decrease in IFP. Therefore normalization enabled the drugs to reach deeper regions of the tumor.

## Methods

### Tumor cells, vasculature and IFP

Following Kohandel *et al*. [46], the Eqs (1) and (2) are used to model the spatio-temporal distribution of tumor cells and the heterogeneous tumor vasculature. In Eq (1), the first term models the diffusion of tumor cells, where *D*_*n*_ is the diffusion coefficient, and the second term describes the tumor growth rate, where *n*_*lim*_ is the carrying capacity and *r* is the growth rate. In the absence of the third and fourth terms, the Eq (1) has two fixed points: an unstable fixed point at *n* = 0 where there is no cell population and a stable fixed point at *n* = *n*_*lim*_ where the population reaches its maximal density. The coupling terms *α*_*mn*_ *n*(**x**, *t*)*m*(**x**, *t*) and *d*_*r*_ *n*(**x**, *t*)*d*(**x**, *t*) indicate the interactions of tumor cells with vasculature and chemotherapy drug, respectively. Tumor cells proliferate at an increased rate *α*_*mn*_ when they have vessels supplying them with nutrients and tumor cells are eliminated at rate *d*_*r*_ if chemotherapy drug *d*(**x**, *t*) is present.

$$\begin{array}{c} \frac{\partial n(x,t)}{\partial t}=D_{n}\nabla^{2}n\left(x,t\right)+rn(1-\frac{n}{n_{lim}})+\alpha_{mn}n\left(x,t\right)m\left(x,t\right)-d_{r}n\left(x,t\right)d\left(x,t\right). \end{array}$$ (1)

The tumor vasculature network exhibits abnormal dynamics with tortuous and highly permeable vessels which are structurally and functionally different from normal vasculature. In order to create this heterogeneous structure, a coarse-grained model is used to produce islands of vessels. In Eq 2, the average blood vessel distribution is represented with m(**x**,t) and the equation is formulated to produce islands of vascularized space with the term *m*(**x**, *t*) (*α* + *βm*(**x**, *t*) + *γm*(**x**, *t*)^2^) which has two stable points *m* = 1 and *m* = 0 corresponding to the presence and absence of vessels, respectively. Representation of tumor-induced angiogenesis is modified in this model by recruiting the terms *α*_*nm*_ *n*(1 − *n*/*n*_*lim*_)*m* and *β*_*nm*_∇.(*m*∇*n*). Here, the former attains positive values for tumor periphery due to the low cell density and in the central regions when cell density exceeds *n*_*lim*_, the term becomes negative creating a behavior which resembles to real tumors that has generally high vascularization in periphery and low vessel density in the center due to the growth-induced stresses [48]. The latter term leads the vessels that are produced in the periphery towards the tumor core. In this novel form, parameters relate to angiogenesis, *β*_*nm*_ and *α*_*nm*_ are changed as 0.5 and 0.25, respectively. Remaining set of the parameters related to tumor and vessel growth can be found in Kohandel et al. [46]. *A*_*r*_ *m*(**x**, *t*)*A*(**x**, *t*) is the reaction of tumor vessels to antiangiogenic agent *A*(**x**, *t*), which results in the elimination of vessels in the presence of antiangiogenic agent.

$$\frac{\partial m(x,t)}{\partial t}=D_{m}\nabla^{2}m\left(x,t\right)+m\left(x,t\right)(\alpha +\beta m\left(x,t\right)+\gamma m{\left(x,t\right)}^{2})+\beta_{nm}\nabla \cdot \left(m\nabla n\right)+\alpha_{nm}n(1-\frac{n}{n_{lim}})m-A_{r}m\left(x,t\right)A\left(x,t\right).$$ (2)

For the initial configuration of tumor cells, a Gaussian distribution is assumed while the initial vascular distribution is obtained by starting from a random, positively distributed initial condition of tumor vessels.

Darcy’s law is used to describe the interstitial fluid flow within the tissue: **u** = −*K***∇***P*, where *K* is the hydraulic conductivity of the interstitium (mm^2^/s/mmHg) and *P* is the interstitial fluid pressure (IFP). For the steady state fluid flow, the continuity equation is:

$$\begin{array}{c} \nabla \cdot u=\Gamma_{b}-\Gamma_{\ell }, \end{array}$$ (3)

where Γ_*b*_ (1/s) represents the supply of the fluid from blood vessels into the interstitial space and Γ_ℓ_ (1/s) represents the fluid drainage from the interstitial space into the lymph vessels. Starling’s law is used to determine the source and the sink terms:

$$\begin{array}{ccc} \Gamma_{b} & = & \lambda_{b}m\left(x,t\right)\;[P_{v}-P\left(x,t\right)-\sigma_{v}\left(\pi_{c}-\pi_{i}\right)], \end{array}$$ (4)

$$\begin{array}{ccc} \Gamma_{\ell } & = & \lambda_{\ell }P\left(x,t\right). \end{array}$$ (5)

The parameters in these equations are the hydraulic conductivities of blood vessels λ_*b*_ and the lymphatics λ_ℓ_, the vascular pressure *P*_*v*_, interstitial fluid pressure *P* and the osmotic reflection coefficient *σ*_*v*_. The capillary and the interstitial oncotic pressures are denoted by *π*_*c*_ and *π*_*i*_, respectively. Hydraulic conductivities of blood and lymph vessels are related to the hydraulic conductivity of vessel wall (*L*_*p*_) and the vessel surface density ($\frac{S}{V}$) with the relation $\lambda_{b,\ell }=L_{p}\frac{S}{V}$. The osmotic pressure contribution for the lymph vessels is neglected due to the highly permeable lymphatics. Also, the pressure inside the lymphatics is taken to be 0 mm Hg [49]. By substituting Darcy’s law and Starling’s law into the continuity equation, we obtain the equation for IFP in a solid tumor:

$$\begin{array}{c} -K\nabla^{2}P\left(x,t\right)=\lambda_{b}m\left(x,t\right)\;[P_{v}-P\left(x,t\right)-\sigma_{v}\left(\pi_{c}-\pi_{i}\right)]-\lambda_{\ell }P\left(x,t\right). \end{array}$$ (6)

Pressure is initially taken to be the normal tissue value *P*_*v*_ and the initial pressure profile is set based on the solution of the above equation with the initial condition for tumor vasculature. The boundary condition ensures that pressure reduces to the normal value *P*_*v*_ in host tissue.

### Antiangiogenic agent and chemotherapy drug

For the transport of antiangiogenic agents *A*(**x**, *t*), a diffusion equation is used:

$$\begin{array}{c} \frac{\partial A(x,t)}{\partial t}=D_{A}\nabla^{2}A\left(x,t\right)+\lambda_{A}m\left(x,t\right)(A_{v}-A\left(x,t\right))-\Gamma_{\ell }A\left(x,t\right)-k_{A}A\left(x,t\right), \end{array}$$ (7)

where *D*_*A*_ is the diffusion coefficient of antiangiogenic agents in tissue, λ_*A*_ is the transvascular diffusion coefficient of antiangiogenic agents, *A*_*v*_ is the plasma antiangiogenic agent concentration and *k*_*A*_ is the decay rate of antiangiogenic agents. The terms on the right hand side represent the diffusion of the antiangiogenic agents in the interstitium, diffusion through the vessels, the drainage of agents to the lymph vessels and the decay rate of the agents, respectively.

We consider liposomal delivery vehicles for chemotherapy drug with their concentration denoted by *d*(**x**, *t*). Since they are relatively large (∼ 100 nm), a convection-diffusion equation is used for the transport of these drug molecules:

$$\frac{\partial d(x,t)}{\partial t}=D_{d}\nabla^{2}d\left(x,t\right)+\nabla \cdot (k_{E}d\left(x,t\right)K\nabla P)+\Gamma_{b}\left(1-\sigma_{d}\right)d_{v}-\Gamma_{\ell }d\left(x,t\right)-d_{r}d\left(x,t\right)n\left(x,t\right)-k_{d}d\left(x,t\right),$$ (8)

where *D*_*d*_ is the diffusion coefficient of drugs in the tissue, *k*_*E*_ is the retardation coefficient for interstitial convection, *d*_*v*_ is the plasma drug concentration, *σ*_*d*_ is the solvent drag reflection coefficient, *d*_*r*_ is the rate of drug elimination as a result of reaction with tumor cells and *k*_*d*_ is the decay rate of the drugs. The terms on the right hand side represent the diffusion and the convection of the drugs in the interstitium, convection of the drugs through the vessels, the drainage of the drugs into the lymphatics, the consumption of drugs as a result of tumor cell interaction and the decay of the drug, respectively. Diffusion of the drug from the blood vessels is assumed to be negligible since transvascular transport of large drugs is convection-dominated.

Since the time scale of the tumor growth is much larger than the time scale for the transport and distribution of the drug molecules, both antiangiogenic agent and chemotherapy drug equations are solved in steady state, i.e. $\frac{\partial d(x,t)}{\partial t}=\frac{\partial A(x,t)}{\partial t}=0$. Both drugs are administered to the plasma with bolus injection in each administration through an exponential decay function:

$$\begin{array}{c} A_{v}\;(t)=A_{0}e^{-t/t_{1/2}^{A}}, \end{array}$$ (9)

$$\begin{array}{c} d_{v}\;(t)=d_{0}e^{-t/t_{1/2}^{d}}, \end{array}$$ (10)

In these equations, the terms *A*_0_, *d*_0_ and $t_{1/2}^{A}$, $t_{1/2}^{d}$ indicate the peak plasma concentration and the plasma half-lives of the antiangiogenic agent and chemotherapy drug, respectively. No-flux boundary conditions are used for the antiangiogenic agent and the chemotherapy drug.

Parameters related to transport of interstitial fluid and transport of liposomes and antiangiogenic agents are listed in Tables 1 and 2 respectively. Some of the effective parameters in the equations above dynamically change to mimic the changes in tumor and its microenvironment. As the tumor grows, lymph vessels are diminished to ensure that there are no lymph vessels inside the tumor. Without the presence of tumor, vessel density can increase up to a specific value (the dimensionless value of 1). When vessel density is greater than 1, it implies that they were produced by angiogenesis and leaky, thus their hydraulic conductivity is increased up to levels that is observed in tumors. During antiangiogenic treatment, vessel density is decreased and when it decreases below 1, normalization occurs and the hydraulic conductivity returns to normal tissue levels.

**Table 1:** Interstitial fluid transport parameters.

Parameter	Unit	Tumor	Normal
[*L*_*P*_]_blood_	cm/mmHg⋅s	1.86×10^−6,a^	3.6×10^−8,a^
${\left[\frac{S}{V}\right]}_{blood}$	cm^2^/cm^3^	200^b^	70^b^
λ_*b*_	1/mmHg⋅s	3.72×10^−4,c^	2.52×10^−6,c^
λ_ℓ_	1/mmHg⋅s	0^d^	6.66×10^−4,e^
*P*_0_	mmHg	15^a^	15^a^
*σ*_*v*_ (*π*_*p*_ − *π*_*i*_)	mmHg	2.2×10^−4,a^	9.1^a^
*P*_eff_ ^f^	mmHg	15	5.9
*K*	cm^2^/mmHg⋅s	2.5×10^−7,a^	2.5×10^−7,a^

^a^ [15],

^b^ [50],

^c^calculated from $L_{P}\frac{S}{V}$,

^d^ [51, 52],

^e^ [36] and

^f^ *P*_eff_ = *P*_*v*_ − *σ*_*v*_ (*π*_*p*_ − *π*_*i*_), effective microvascular pressure.

**Table 2:** Parameters related to transport of 100 nm liposomes and antiangiogenic agents.

Parameter	Unit	Tumor	Normal
*D*_*d*_	cm^2^/s	2.5×10^−9,a^	2.5×10^−9,a^
*k*_*E*_	-	0.35^b^	0.35^b^
1 − *σ*_*d*_	-	0.87^c^	0
*P*_*d*_	cm/s	5%×3.42×10^−7,d^	5%×0.88×10^−7,d^
λ_*d*_	1/s	4×10^−6,e^	1.4×10^−7,e^
λ_*r*_	1/hours	1/135^f^	1/135^f^
$t_{1/2}^{d}$	hours	45.2^g^	45.2^g^
*d*_*r*_	1/hours	1	0
*k*_*d*_	1/s	1.65×10^−6^	1.65×10^−6^
*D*_*A*_	cm^2^/s	4×10^−7^	4×10^−7^
λ_*A*_	1/s	4×10^−6,g^	1.4×10^−7^
*A*_*r*_	1/hours	1	1
*k*_*A*_	1/s	1.65×10^−6^	1.65×10^−6^
$t_{1/2}^{A}$	hours	20	20

^a^ [53]

^b^ estimated from [54]

^c^ estimated using *σ*_*d*_ = (1 − (1 − *α*)^2^)^2^ with ratio of drug to pore radius, *α* = 100 nm/500 nm [55]

^d^ diffusional permeabilities taken to be %5 of the effective permeabilities measured in [56]

^e^ calculated using $P_{d}\frac{S}{V}$

^f^ [57]

^g^ [58]

## Results

**Fig 1:**
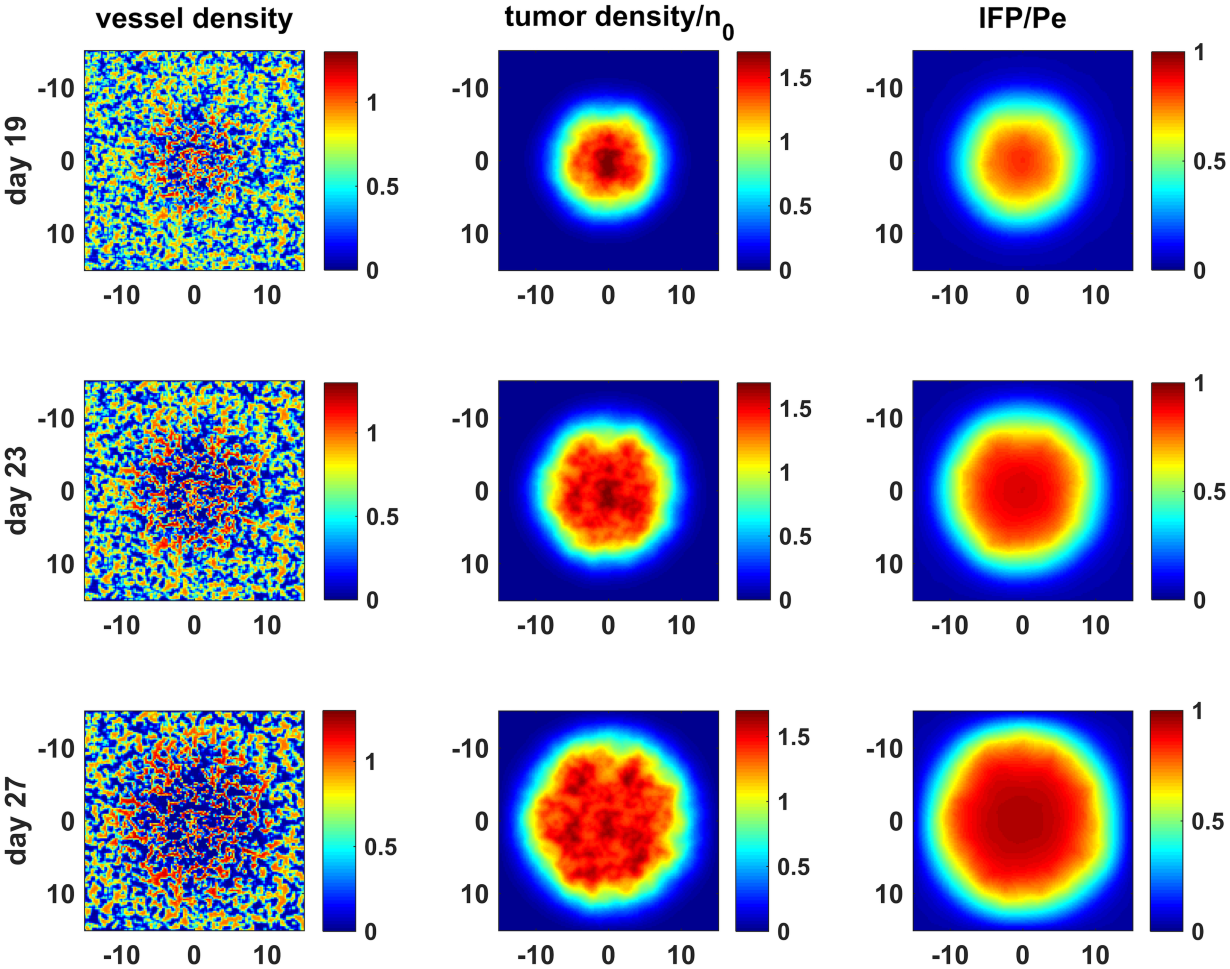
Tumor growth for control case. Vessel and tumor densities and IFP for days 19, 23 and 27.

We started the simulations with a small tumor (0.2 mm radius) and left it to grow for 30 days to an approximate radius of 13.5 mm. Vessels which were initially set as randomly distributed islands in the computational domain evolved into a heterogeneous state throughout the simulations due to the presence of tumor cells (Fig 1, vessel density). As the tumor grows, vessel islands become sparse in the interior region but their density increases by angiogenesis and they become leaky. By the end of the simulation, the leakiness of tumor vessels and the lack of lymphatic drainage inside the tumor causes elevated pressure in the interior region of tumor very similar to that suggested in literature [1, 5] (Fig 1, IFP/Pe).

**Fig 2:**
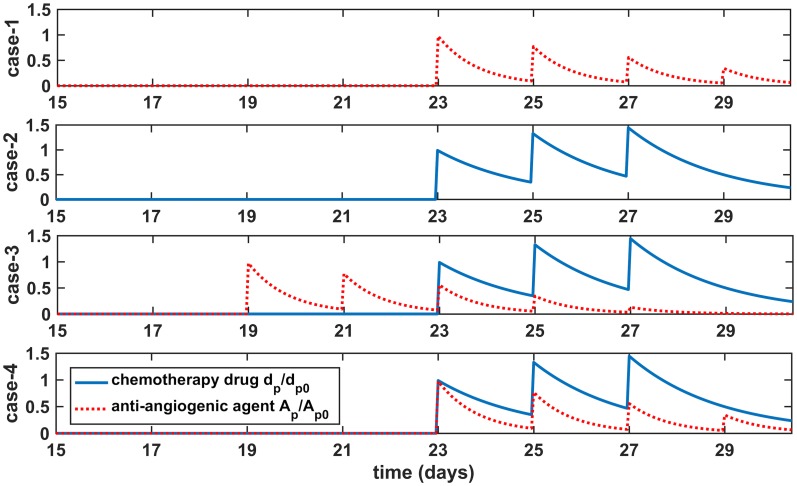
Drug regimens. From top to bottom, regimens for antiangiogenic agents alone (case-1), chemotherapy alone (case-2) and the combined therapy of antiangiogenic agents and chemotherapy drug (case-3 and case-4) which show plasma concentrations for each drug over time.

We experimented with various drug regimens. To illustrate the improvement in drug delivery, we designed the cases given in Fig 2. Dimensionless dose values are fixed in order to replicate the treatment response observed in [47]. Antiangiogenic treatment is adjusted such that at the end of administrations there is approximately a 50% decrease in MVD inside the tumor. A fixed chemotherapy drug dose is administered on days 23, 25 and 27 while we change the day of antiangiogenic agent administration starting from the days 15, 17, 19, 21 and 23, continue to give them every other day in 4 or 5 pulses. We decrease the dose of antiangiogenic agents throughout the therapy because a better response in drug delivery is obtained with this way in our simulations. We present here four cases where only antiangiogenic agent administration starts on day 23, only chemotherapy drug on day 23, neoadjuvant therapy with antiangiogenic agents on day 19 and chemotherapy drug on day 23 and finally concurrent therapy with both of drugs starting on day 23. The most beneficial results regarding the amounts of drugs extravasate in the interior parts of the tumor are yielded when the antiangiogenic treatment starts at day 19 (case-3 in Fig 2).

**Fig 3:**
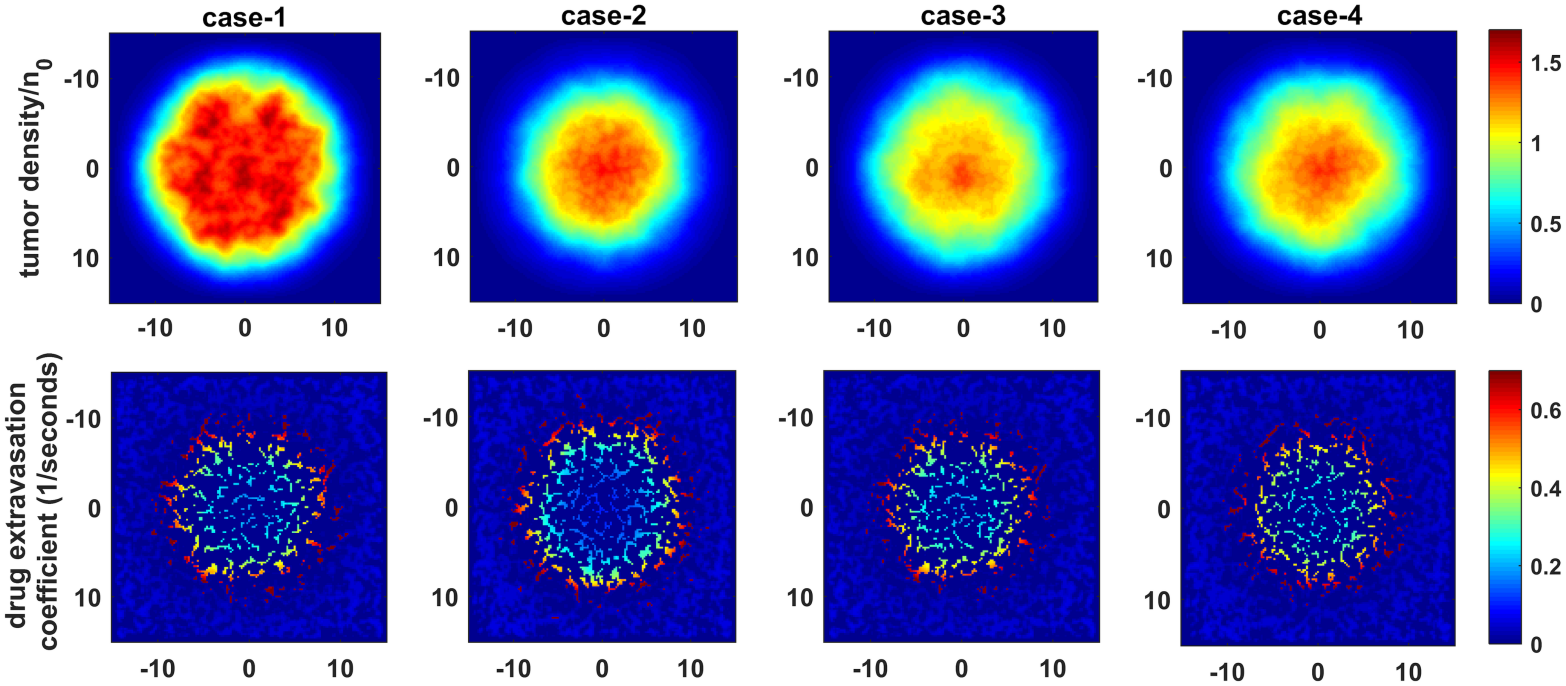
Tumor density and chemotherapy drug extravasation coefficient. Tumor density and chemotherapy drug extravasation coefficient (Γ_*b*_(1 − *σ*_*d*_)) are calculated for day 27 which corresponds with last application chemotherapy drug.

**Fig 4:**
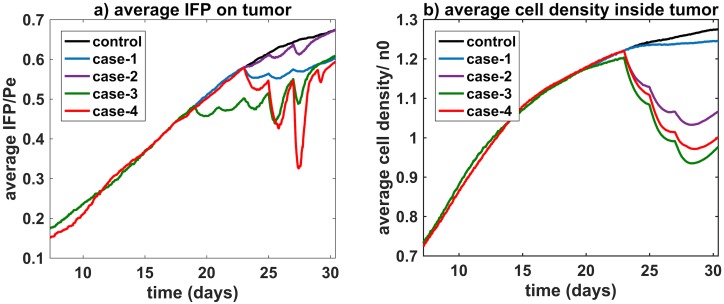
Average IFP over whole tumor and average cell density in the interior region of tumor. Eqs 12 an 11 are used to calculate average IFP and average cell density, respectively.

As expected, antiangiogenic agents don’t have a profound effect on tumor cell density when they are applied alone (Fig 3, case-1). In all cases, we observed greater drug extravasation near the tumor rim due to decreasing IFP in that region (Fig 3). It can be seen that fluid flow from vessels to the tumor is poor in the interior region for case-2, but it starts to enhance in the same region in case-3 and case-4. The main reason for this change is the introduction of a pressure gradient in the tumor center restoring drug convection. Therefore, in both case-3 and case-4, tumor cell density is decreased in the interior region (Figs 3 and 4b) as a consequence of increased drug extravasation in the interior region of the tumor.

We calculate the space average of cell density and IFP in each time step. Average cell density is calculated as

$$\begin{array}{c} \frac{{\;\int \int \;}_{A_{int}}\;n\left(x,y,t\right)\;dx\;dy}{{\;\int \int \;}_{A_{int}}\;dx\;dy} \end{array}$$ (11)

over area *A*_*int*_ whose boundary is set by the condition *n*(**x**, **y**, *t*) > 1 which represents the interior region of tumor (corresponds to *r* < 6 mm for a tumor of radius 10mm). Average IFP is calculated as

$$\begin{array}{c} \frac{{\;\int \int \;}_{A}\;P\left(x,y,t\right)\;dx\;dy}{{\;\int \int \;}_{A}\;dx\;dy} \end{array}$$ (12)

over area *A* whose boundary is set by the condition *n*(**x**, **y**, *t*) > 0.1 which represents the value over whole tumor.

When we evaluate average pressure over the entire area of the tumor, we observe a synergistic effect in reducing pressure arising from the combined application of antiangiogenic agent and chemotherapy which can be seen in Fig 4a, especially for case-4. This synergistic effect also exhibits itself in tumor cell density in a less pronounced manner that can be observed from Fig 4b. This indicates improved combination treatment efficacy as an indirect result of decreasing IFP.

**Fig 5:**
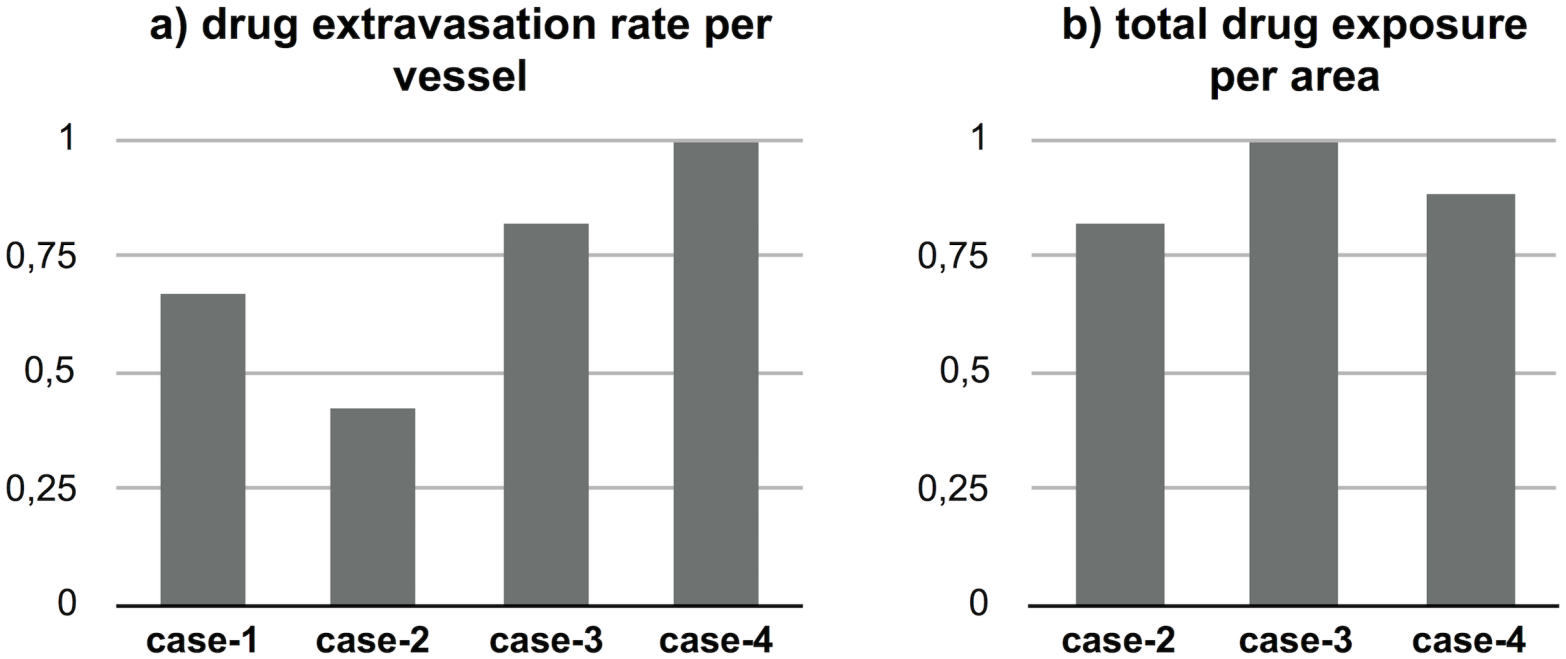
Drug extravasation rate from vessels and total drug exposure per unit area in the interior region of tumor by the end of the simulation.

According to our results, drug extravasation from vessels in the interior region of the tumor is nearly doubled for combination cases (Fig 5a, case-3 and case-4 compared to case-2). However, this improvement is not directly reflected on drug exposure due to reduced vessel density by antiangiogenic agents. Total drug exposure of unit area in tumor during treatment only improves approximately 20–25%.

IFP during the applications of chemotherapy drug was the lowest for concurrent therapy (case-4). However, regarding tumor regression adjuvant therapy (case-3) performed better, agreeing with the results of Kohandel et al. [46].

Even though decrease in vessel density and leakiness cuts off the supply of drugs, the decrease in IFP appearing for the same reasons seems to compensate in the interior region of tumor, resulting in better drug extravasation. When two drugs are given closer temporally, the resulting IFP decrease is maximized. This enables the convective extravasation of nanoparticles deep into tumors to places that are not exposed to drugs without combination therapy.

**Fig 6:**
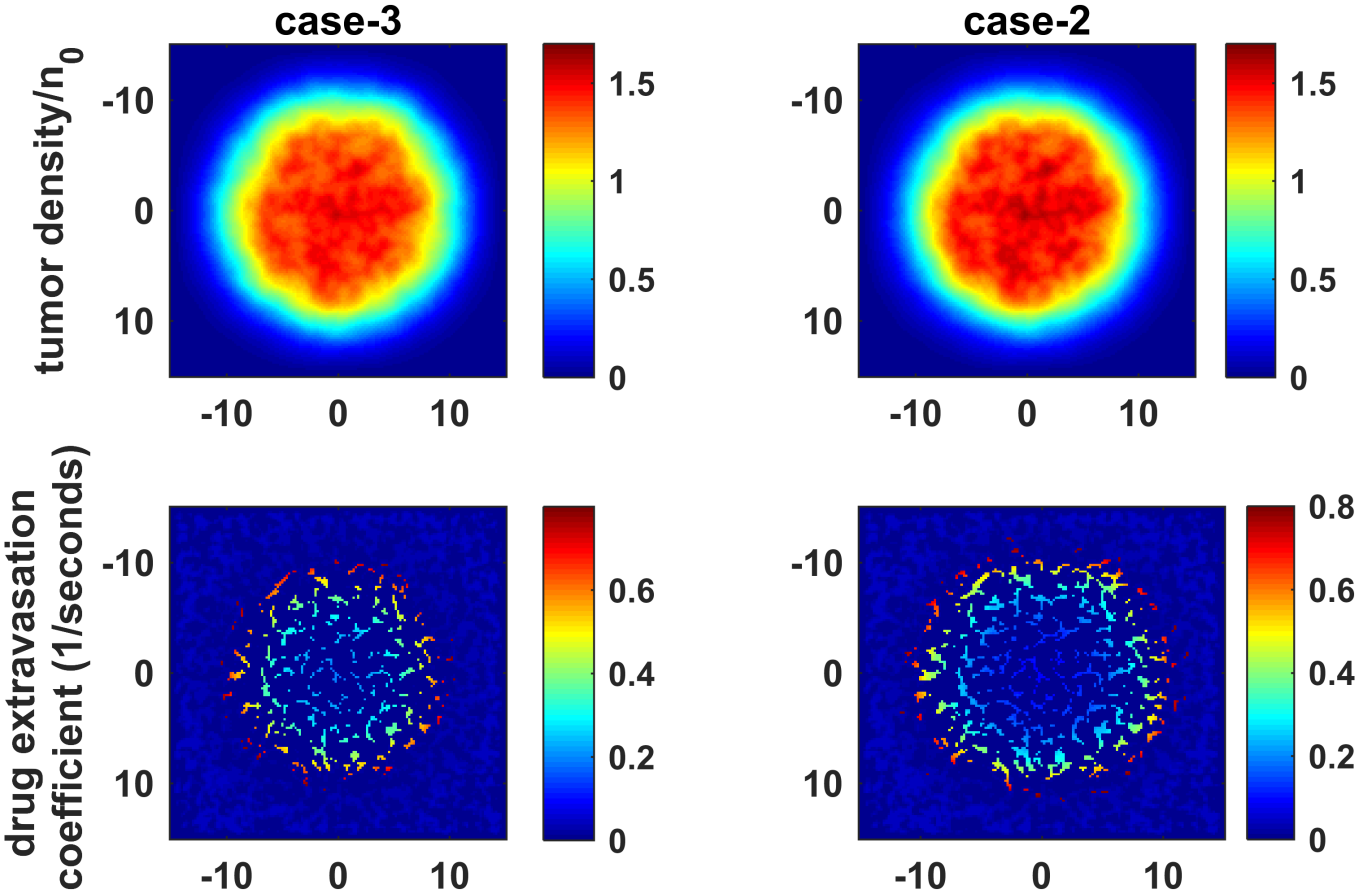
Tumor density and chemotherapy drug extravasation coefficient. Tumor density and chemotherapy drug extravasation coefficient (Γ_*b*_(1 − *σ*_*d*_)) are calculated for day 27 which corresponds with the last application of chemotherapy drug for the chemotherapy drugs that inhibit tumor growth.

In order to evaluate the effect of chemotherapy drugs that target tumor cell proliferation, we modified Eq 1 such that the chemotherapy drugs would directly act on tumor growth. The terms responsible for tumor growth (2nd and 3rd terms in the right-hand side of Eq 1) are multiplied by (1 − *d*(*x*, *t*)/*d*_*max*_) where *d*_*max*_ is maximum drug concentration that extravasated inside the tumor. In this scenario, small changes are seen in tumor cell densities between combination therapy and chemotherapy alone. However, we observe that in this form, extravasation of drugs is also increased in the central region as seen in Fig 6, implying that normalization is also beneficial in this scenario.

## Discussion

Using a mathematical model, we assess whether antiangiogenic therapy could increase liposome delivery due to normalization of tumor vessels. In order to do that, we first created a dynamic vessel structure that exhibits properties of tumor vessels created by angiogenesis as well as inherent vessels in the tissue. As the tumor grows, vessels in the central region begin to disappear due to increased tumor cell density in that region. Angiogenesis occurs in the tumor creating additional leaky vessels. The emergent vessel density is consistent with that observed in [59], with decreasing density towards the tumor center along with randomly appearing clusters of vessels. IFP is found to be elevated throughout the tumor up to the levels of MVP and decreases sharply around the tumor rim as it is observed in various studies in the literature. [1, 5, 6].

We apply antiangiogenic agents in various regimens combined with chemotherapy and focus on large drugs (liposomes) whose delivery mainly depends on convection. As a result of the decrease in vessel density and leakiness due to the antiangiogenic activity, we expect a decrease in pressure which brings about a higher pressure difference between tumor and vessels. Transvascular convection depends on this pressure difference, hydraulic conductivity and density of vessels at the unit area. Since antiangiogenic agents decrease hydraulic conductivity (i.e. leakiness) and vessel density, by cutting the supply of drugs, the resulting increase in pressure difference should compensate for these effects, restoring extravasation in remaining vessels.

In all simulations, liposome extravasation predominantly occurs in the tumor periphery due to low IFP levels, hence drugs preferentially accumulate in this area. Our result has been confirmed by experimental studies of drug distribution using large drugs such as micelles [60, 61], nanoprobes [62] and liposomes [59, 63–66] in which peripheral accumulation is observed.

As the application time between antiangiogenic agents and liposomes becomes shorter, the resulting decrease in IFP is maximized. This enables the convective extravasation of nanoparticles deep into tumors to places that could not previously be exposed to drugs before and liposome extravasation begins to appear in central region. However, that does not bring about maximum accumulation of liposomes consistently at all times. There is a trade-off between total drug accumulation and how deeply drug can penetrate inside the tumor. In our study, we find a balance between these two situations. It also shows us that IFP and drug accumulation are not always correlated, rather the maximum accumulation is achieved through the complex interplay between IFP, vessel density and leakiness. Current research by [63] also supports this view; in their mouse study, they point out that IFP is correlated with perfusion, perfusion is correlated with accumulation and the relationship between IFP and liposome accumulation is limited.

In another significant study, tumor-bearing animals are subjected to combination therapy with liposomes and the antiangiogenic agent pazopanib in order to evaluate the effect of normalization via imaging drug distribution [65]. As a result of the decrease in MVP, they also observed a resulting decrease in IFP. Similar to our results, IFP is not the determinant of drug accumulation in their work. They have found that decreased leakiness of vessels inhibits delivery even though there is an IFP decrease as a result of antiangiogenic therapy. They have collected data for a single time point and observed a decrease in doxil penetration in combination therapy. They also point out that functional measures of normalization may not occur simultaneously which is also the case for our study. Throughout the combination therapy, we also observe periods where drug extravasation is limited and others where drug extravasation is improved. They have found the vessel permeability as a limiting factor in their study, however MVD [67] and tumor blood flow and blood volume [68] are also determinants of large drug accumulation. This shows that these measures of normalization are tumor type dependent and even within the same tumor they are dynamic which leads to variation in drug distribution.

Among many different schedules, most of our trials did not show improvement in drug accumulation. We see that the dose of antiangiogenic agents should be carefully determined to ensure any delivery benefit. As stated by [30], when we apply a large dose of antiangiogenic agents, significant IFP decrease is observed but the decrease in vessel permeability and the lack of vessel density lead to impaired liposome extravasation. At the other extreme, when we give small amounts of ant-angiogenic agents, it is seen that IFP decrease is not enough to make a significant improvement to liposome extravasation.

In this model, intravascular flow is approximated as uniform to focus on the effects of transvascular delivery benefit of normalization. Due to abnormal vasculature, tumors are known to have impaired blood perfusion [69] due to simultaneous presence of functional and non-functional vessels. In this work, we simulate structural normalization of vessels without considering functional normalization which is associated with intravascular flow and results in increased perfusion [30]. Vessels within the tumor in this model have uniform functionality in terms of supplying blood flow. Hence, by decreasing vessel density in microenvironment due to antiangiogenic activity, we are decreasing blood perfusion. However, on the contrary, normalization is expected to enhance intravascular flow by decreasing pore size which restores intravascular pressure gradients and pruning non-functional vessels that interrupt circulation. Therefore, normalization brings about improved blood perfusion whereas here we decrease perfusion and improve the delivery only through improved convective extravasation by decreased IFP. In our simulations, the delivery benefit is underestimated since we decrease blood perfusion as a part of antiangiogenic activity. In [65], they observed that MVD decrease did not change liposome accumulation because the eliminated vessels are the ones that are thought to be nonfunctional. In our previous study, we constructed a spherical tumor with uniform vessel density to investigate the benefit from normalization therapy and the results showed increased delivery in the interior regions of tumors of certain sizes [70].

In animal studies, it has been shown that the bulk accumulation of liposomes is not representative of efficacy since it is not informative about the drug accumulation within specific regions of tumors [67, 71] and heterogeneous drug accumulation may result in tumor repopulation [72]. Therefore, it is important to understand the factors that yield heterogeneous accumulation and strive to avoid them to generate effective treatments.

According to our results, it is plausible that administering targeted therapies using large drugs, normalization should be more useful since it can provide a simultaneous access to both tumor rim and center. The dose of chemotherapy should be increased in order to ensure similar drug exposure despite the sparser vessel density caused by antiangiogenic activity. This is the reason why targeted therapies are more suitable to seize the benefits from normalization, as they can be applied in greater doses without harming healthy tissue. When convective extravasation is restored in the central region, drugs can immediately reach to tumor center and increase the probability of treatment success and tumor eradication.
